# Force-Displacement Analysis in Diaphragm-Embedded Fiber Bragg Grating Sensors

**DOI:** 10.3390/s22145355

**Published:** 2022-07-18

**Authors:** Arnaldo Leal-Junior, Vitorino Biazi, Carlos Marques, Anselmo Frizera

**Affiliations:** 1 Mechanical Engineering Department, Federal University of Espírito Santo, Vitória 29075-910, Espirito Santo, Brazil; 2 Graduate Program in Electrical Engineering, Federal University of Espírito Santo, Vitória 29075-910, Espirito Santo, Brazil; vitorino.biazi@edu.ufes.br (V.B.); frizera@ieee.org (A.F.); 3 Department of Physics and I3N, Campus Universitário de Santiago, University of Aveiro, 3810-193 Aveiro, Portugal; carlos.marques@ua.pt

**Keywords:** fiber Bragg gratings, force sensors, viscoelasticity, polymers

## Abstract

This paper presented the force and displacement analyses of a diaphragm-embedded fiber Bragg grating (FBG) sensor. In the first step, a numerical analysis (via finite element method) was performed considering linear elastic materials, where there is a linear variation on the strain in the optical fiber for both displacement and force (or pressure). In the second step, the experimental analysis was performed using two approaches: (i) controlling the displacement applied in the diaphragm-embedded FBG (while the force is also measured). (ii) Controlling the force applied in the sensor (also with the measurement of the displacement). Results showed reflected optical power variations and wavelength shift following the application of displacement and force. The sensitivities of both wavelength shift and optical power were different (and non-proportional) when displacement and force were compared. However, a higher correlation, determination coefficient (R^2^) of 0.998, was obtained in the analysis of the wavelength shift as a function of the displacement, which indicated that the strain transmission in the optical fiber is directly related to the strain in the diaphragm, whereas the force has an indirect relation with the strain and depends on the material features. Then, the possibility of simultaneous estimation of force and displacement was investigated, where the linear relation of both parameters (displacement and force) with the wavelength shift and the optical power were obtained in a limited range of displacement and force. In this range, root mean squared errors of 0.37 N and 0.05 mm were obtained for force and displacement, respectively. In addition, the force variation with a step displacement input also shows the possibility of using the proposed FBG device for the characterization of the materials’ viscoelastic features such as phase delay, creep, and stress relaxation, which can be employed for in situ characterization of different viscoelastic materials.

## 1. Introduction

Optical fiber sensors experienced a continuous widespread over the years from the first developments restricted to laboratory environments to practical applications in which the optical fiber-based sensor systems present important advantages over conventional measurement techniques, e.g., electronic and electromechanical systems. These advantages include the galvanic isolation, corrosion resistance, compactness, remote sensing operation, multiplexing capabilities, and insensitivity to electromagnetic interferences [1]. Although these advantages enabled some niche applications, especially in harsh environments [2] and for long-range sensing [3], the constant developments on optical fiber sensing technologies, especially on portable devices and lower-cost sensing techniques [4], lead to their applications in a multitude of applications, which include smart textiles [5], biomechanics [6], medical devices [5], structural monitoring [7], and immunosensing [8].

One of the most popular optical fiber sensing techniques is the fiber Bragg gratings (FBGs), which are created through a periodic modulation on the optical fiber core’s refractive index [9]. In their first reports decades ago, FBGs were used as filters and dispersion compensation components in optical communications [10]. Over the years, the use of FBGs as sensors have been proposed due to the grating tunability with strain and temperature [11]. The use of FBGs in sensors applications is motivated by the combination of accuracy and customizability due to the wavelength-encoded data (which make the FBGs insensitive to light source power deviations) and the multiplexing capabilities, where multiple FBGs can be inscribed in a single optical fiber cable by changing only the period in the refractive index modulation [12]. In addition, new developments in portable interrogation equipment [13] and low-cost interrogation approaches [14] resulted in a larger widespread of FBGs in different applications [15].

The optical fiber properties enable their embedment in different materials and structures, which in conjunction with the FBGs’ advantageous features resulted in the embedment in mechanical elements [16], cantilevers [17], 3D-printed structures [18], and diaphragms [19]. The FBG embedment in different materials can be employed for extending the sensor performance such as increase of sensitivity or dynamic range as well as FBG applications on the assessment of additional parameters, namely pressure [20], flow [21], liquid level [22], and humidity [23].

The FBG embedment in different materials also leads to additional design parameters to be analyzed and optimized according to the desired application, as the geometry parameters (e.g., thickness, diameter, or fiber orientation) can influence the sensor’s performance parameters such as sensitivity, linearity, dynamic range, and hysteresis [24]. Furthermore, the embedment material thermal and mechanical properties influence the sensor responses. In the diaphragm-embedded FBG force or pressure sensors, the sensitivity is related to the material’s elastic modulus, since the sensor is based on the stress/strain transmission between the diaphragm and the optical fiber sensor. For this reason, the sensor modeling considers the material properties and geometries. Commonly, the diaphragms in which the FBGs are embedded are made of rubbers (and polymers, in general), since they can provide a combination of high sensitivity and dynamic range due to their high strain limits and low elastic modulus [24]. Moreover, polymers have viscoelastic response, which is characterized as a non-constant relation between stress and strain [25]. However, a somewhat unexplored feature in these applications is the use of the FBG to evaluate the material properties, since the silica optical fiber does not present a large viscoelastic response at room temperature conditions. In addition, the analysis of additional spectral features of the FBGs (instead of only wavelength shift) enables the possibility of measuring multiple parameters using a single optical fiber [26].

Aiming at this background, this paper presented the force and displacement relation analysis on a diaphragm-embedded FBG sensor, where the variations on the spectral features as a function of force and displacement (or stress and strain) presented a direct relation with the diaphragm material features. To that extent, it is possible to use the FBG as a tool for the analysis of the embedment material as well as the possibility of measuring force and displacement (or strain and stress) in a limited range using FBG sensors.

## 2. Theoretical Background

As thoroughly discussed in the literature and well-explored in the last few years [27], the wavelength shift on FBGs is related to the changes on the refractive index and grating period, which are related to the temperature and strain, as shown in Equation (1).

(1) $$\frac{\Delta \lambda_{B}}{\lambda_{B}}=(1-P_{e})\varepsilon +(\alpha +\zeta )\Delta T$$

where Δ*λ_B_* and *λ_B_* are the Bragg wavelength shift and initial wavelength, respectively. In addition, *P_e_* is the effective photoelastic constant that considers the changes in the optical fiber’s refractive index as a function of the strain (*ε*), where the latter also leads to a variation on the grating period. The right-hand side of the sum is related to the temperature influence on the Bragg wavelength. In this case, the temperature variation (Δ*T*) results in refractive index changes proportional to the thermo-optic coefficient (*ζ*). Furthermore, the temperature leads to a thermal expansion of the optical fiber, also proportional to the fiber material’s thermal expansion coefficient (*α*). The tests presented in this paper were performed at constant temperature conditions. Thus, the temperature influence was not considered in the following analyses.

**Figure 1:**
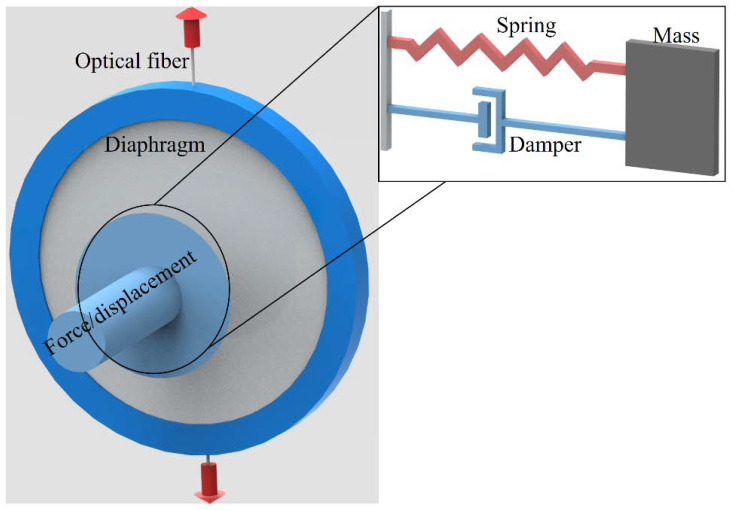
Schematic representation of force transmission. Figure inset shows the spring–mass–damper representation.

As we investigate the influence of displacements and forces inputs on the diaphragm-embedded FBG, the diaphragm properties are also considered in the analysis. In this case, a rubber diaphragm was used, which presents a viscoelastic response represented by a non-constant relation between stress and strain, as discussed in ref. [28]. Among many models of viscoelastic materials, the Maxwell’s model relates the elastic and viscous components of the materials’ responses with spring (for the elastic component) and dampers (for the viscous component) [25]. For this reason, there is a non-constant relation between the stress and strain applied on the diaphragm, especially when the dynamic responses are considered. In addition, the force or displacement applied on the diaphragm are transmitted to the optical fiber, as shown in Figure 1, where a transversal input in the diaphragm generates strain components on the longitudinal axis of the optical fiber. Such stress/strain transmissions are proportional to the materials’ (diaphragm and optical fiber) Poisson’s ratio. Moreover, the spring–damper representation of the diaphragm material (following Maxwell’s model) is presented in Figure 1 inset.

Following the representation in Figure 1, the application of displacement and force on the diaphragm can lead to different displacements (or strain) transmitted to the optical fiber. A simplified and one-degree-of-freedom approach indicates variations differential equations when the force or displacement are considered, represented as Equations (2) and (3) for force and displacement, respectively. Such equations indicate the possibility of differences on the dynamic responses of the material when the displacement and force are considered.

(2) $$m\frac{d^{2}y}{dt^{2}}+b\frac{dy}{dt}+ky=F$$

(3) $$m\frac{d^{2}y}{dt^{2}}+b\frac{dy}{dt}+ky=b\frac{dx}{dt}+kx$$

where *x* and *F* are the displacement and forces applied on the diaphragm, respectively. Similarly, *y* is the displacement on the diaphragm after the force and/or displacement input. The diaphragm assembly and material parameters are represented by *m*, *b*, and *k*, which represents the system’s mass, damping, and stiffness constants, respectively.

It is worth noting that the force or displacements applied on the diaphragm (and transmitted to the FBG) also lead to a bending on the optical fiber. As discussed and modeled in ref. [29], the bending on the fiber results in an attenuation on the reflected optical power. This feature is commonly used on the development of intensity variation-based sensors [30]. Therefore, not only the wavelength shift is analyzed as a function of the applied displacement or force, but also the reflected optical power. Such combined analysis leads to the possibility of simultaneous assessment of multiple parameters as demonstrated in ref. [31]. Furthermore, the FBG position on the diaphragm influences not only the fiber bending (which can lead to the optical attenuation), but also the strain in the fiber. Thus, the numerical and experimental analyses are performed considering the FBG at the center of the diaphragm considering front and side planes.

However, in this case, the analysis of spectral variations on the FBG as a function of the applied displacement and force can also result in the possibility of assessing the viscoelastic features as well as the material properties of the diaphragm. If the dynamic Young’s modulus of the viscoelastic material is considered, there are viscous and elastic components (as also represented in Figure 1), which can be estimated from the force and displacement responses in the sensors.

## 3. Experimental Setup

A numerical analysis for the stress and strain transmission in diaphragm and optical fiber interfaces was performed through the finite element method (FEM) using the Ansys Workbench R17.2 for structural analysis. In this analysis, a cylindrical support was applied on the diaphragm, whereas displacement and forces were transversally applied on the diaphragm. The strain on the embedded optical fiber was analyzed for each condition, where displacements from 2 mm to 5 mm were applied in 1 mm steps, which is the range for the experimental tests, limited by the material’s strain limits and compression machine resolution. In addition, the force was correlated with the pressure, as it involves the force application in the diaphragm area, which results in pressures from 1.5 kPa to 3.0 kPa in 0.5 kPa steps.

For the experimental analyses, an FBG was inscribed in a photosensitive single-mode silica fiber GF1B (ThorLabs, Newton, NJ, USA) via phase-mask technique using a nanosecond pulsed laser at 266 nm LS-2137ULaser (LOTIS TII, Minsk, Belarus). Then, the fiber was embedded in an acrylonitrile butadiene rubber (NBR) diaphragm an oil-resistant synthetic rubber with static Young’s modulus of around 360 MPa. The optical fiber embedment was performed by gluing the fiber into two sheets of diaphragm with 40 mm diameter and 1 mm thickness. Thereafter, a cylindrical support was designed for the diaphragm-embedded FBG, where 3D-printed supports were positioned on the diaphragm in order to enable similar conditions as the ones employed in the numerical simulation.

**Figure 2:**
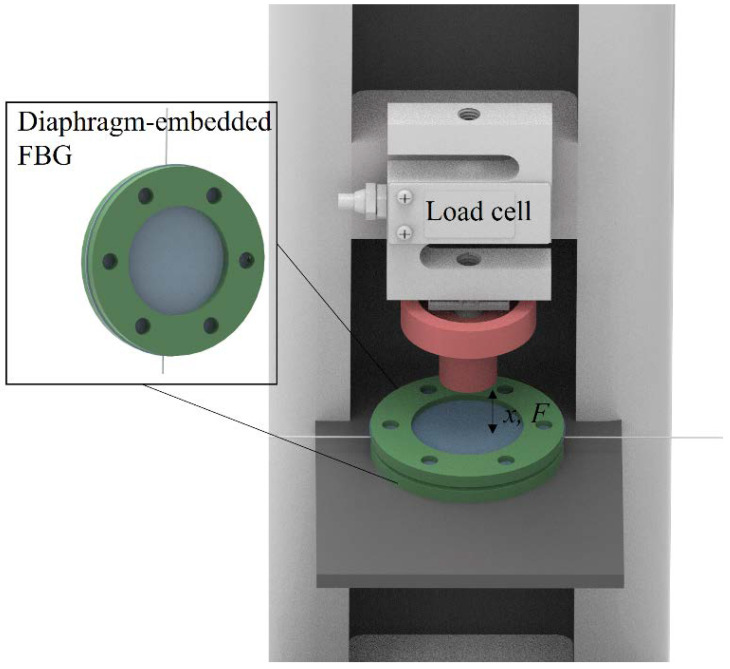
Experimental setup for force and displacement assessment. Figure inset shows the diaphragm structure.

Figure 2 presents the experimental setup for the force and displacement evaluations, where Figure 2 inset presents the schematic representation of the diaphragm structure. In this case, the diaphragm (with embedded optical fiber) structure is positioned on a compression machine comprised of a strain gauge and a displacement sensor to evaluate the wavelength shift and reflected optical power variations as a function of the applied displacement and force. The FBG-reflected spectra and their features (Bragg wavelength and reflectivity) were acquired with the optical interrogator sm125 (Micron Optics/Luna, USA). For the displacement assessment, 5 loading/unloading cycles were performed in the range of 0 to around 3 mm, whereas other 5 tests were performed in the range of 0 to around 13.5 N for the evaluation of the force influence on the sensors responses.

The reflected optical spectra as well as the Bragg wavelength and optical power were analyzed as a function of the applied displacement and force. In the proposed approach, the correlation between the spectral features and force is compared with the correlation of the sensor responses and the displacement. Such comparison enables verifying if the FBG response has higher correlation with force or displacement, which is an important analysis to understand the strain/stress transmission mechanisms of the diaphragm to the optical fiber. Furthermore, the FBG responses on loading and unloading cycles of both displacement and force can also indicate the phase shift between the stress and strain, which is related to the elastic and viscous components of the diaphragm material responses, as discussed in Section 2.

## 4. Results and Discussion

**Figure 3:**
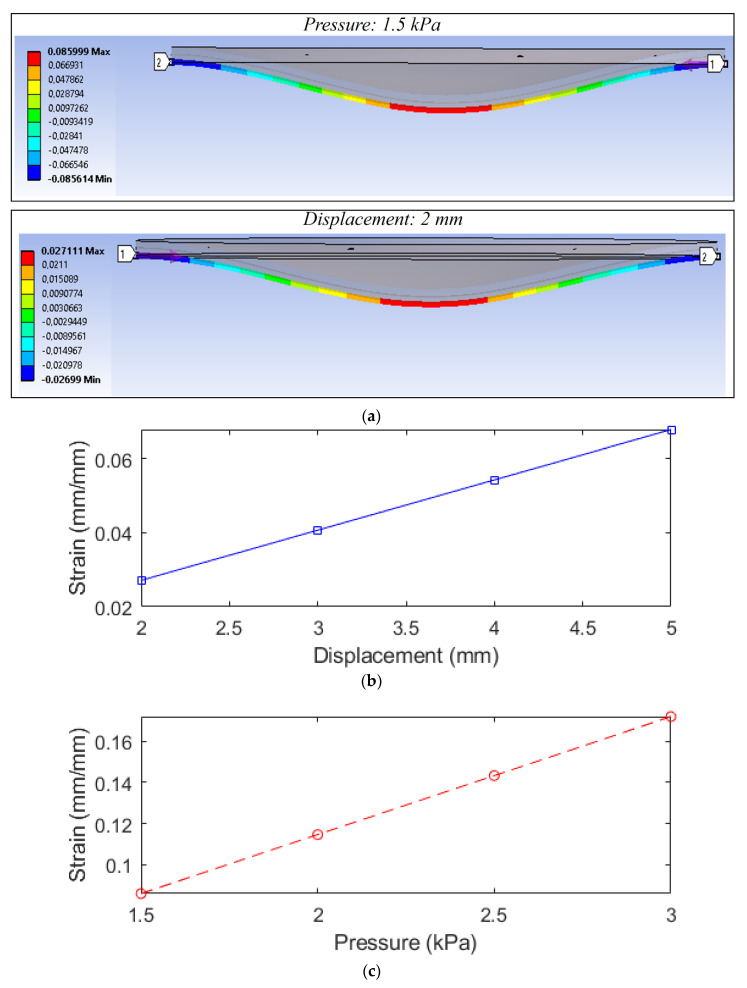
(**a**) FEM results. Strain in the optical fiber as a function of the (**b**) displacement and (**c**) pressure.

Figure 3 presents the results obtained in the FEM simulation in which it is possible to observe the strain distribution along the optical fiber when a displacement and/or force are applied. In addition, Figure 3 presents the strain on the optical fiber as a function of the applied displacement and force (or pressure), where we inferred the differences on the strain transmitted to the optical fiber for each condition. However, both conditions presented a linear response due to material features considered in the numerical analysis in which the viscoelastic effects are neglected. Such difference in the strain behavior can also indicate variations in the spectral features of the FBG, namely wavelength shift and reflected optical power when displacement and forces are applied, especially when a viscoelastic material is considered.

**Figure 4:**
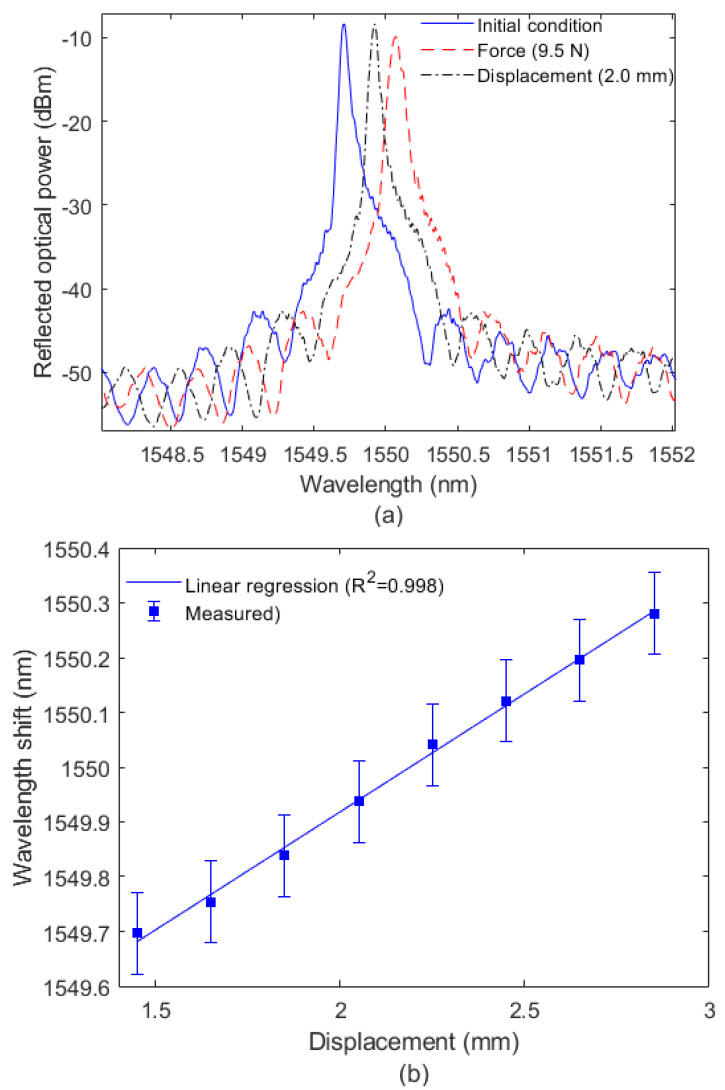

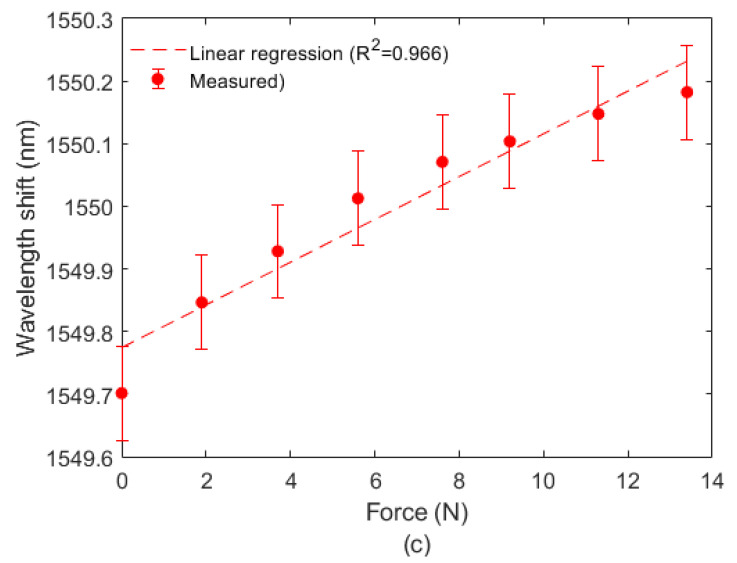
(**a**) FBG reflected spectra in three conditions; (**b**) wavelength shift as a function of (**b**) displacement and (**c**) force.

**Figure 5:**
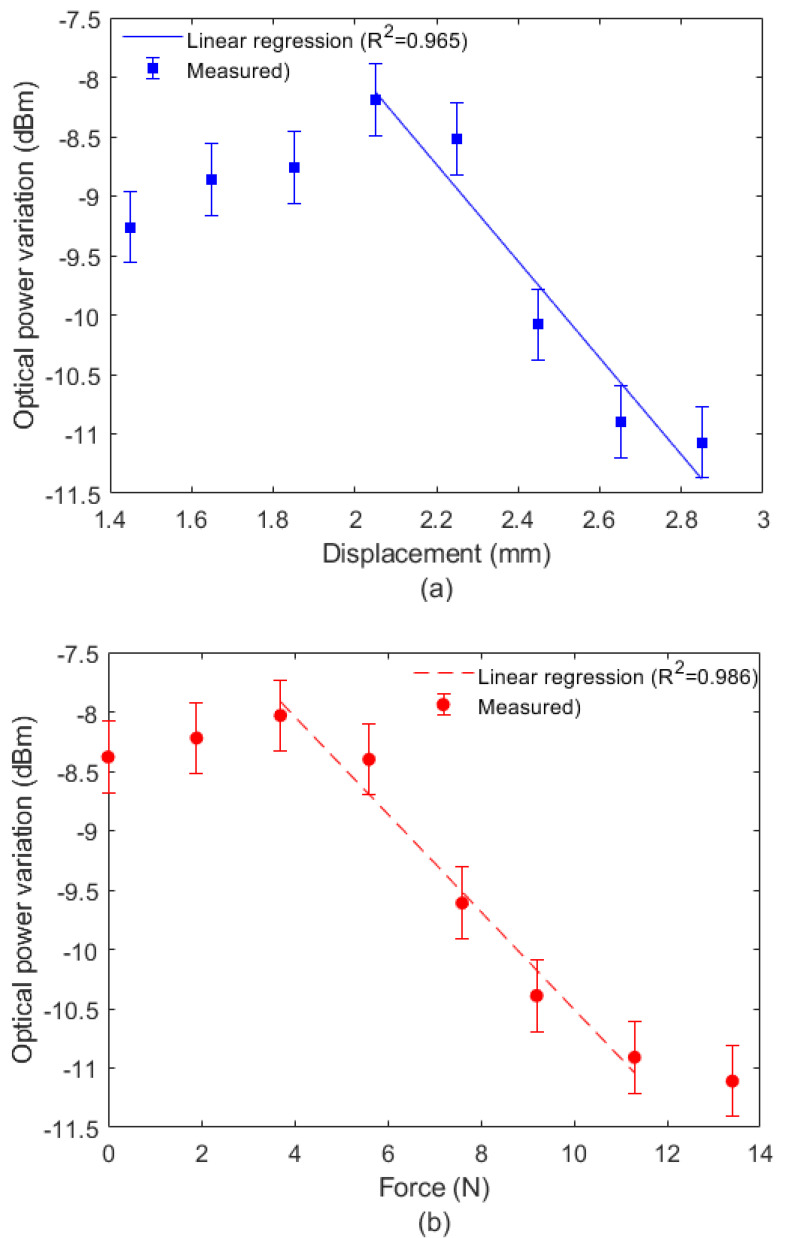
Reflected optical power variation as a function of (**a**) displacement and (**b**) force.

In order to verify the spectral differences in the optical fiber for each condition, Figure 4a presents the FBG’s reflected spectra for three conditions: (i) without strain or stress, (ii) with a controlled displacement, and (iii) with controlled force. Although the differences in the reflected spectra are mainly related to the amplitudes of applied forces and displacements, the analysis of each condition confirmed the hypothesis that the force and displacements transversally applied on the diaphragm lead to both wavelength shift and optical power attenuation. The wavelength shifted as a function of different applied displacements and forces, presented in Figure 4b,c, respectively. Figure 5a,b show the reflected power variations as a function of the displacements and forces on the diaphragm.

The results in Figure 4b,c indicate different sensitivities of wavelength shift for the displacement and force. When the wavelength shift was analyzed, sensitivities of 431.2 pm/mm and 34.1 pm/N were obtained for displacement and force, respectively. The results in Figure 4 also show the determination coefficient (R^2^) in the regression between wavelength as function of force (and displacement).

Similarly, as shown in Figure 5a,b, the reflected optical power also presented different sensitivities to displacement and force, where −3.3 dBm/mm and −0.41 dBm/N were estimated for displacement and force, respectively. However, the optical power variation presented a linear response only in a limited range between around 2.0 mm and 2.8 mm for the displacement, shown in Figure 5a, whereas the range of linear response for force detection was around 4.0 N to 11.0 N. It is worth noting that there is another region with a linear range, from 1.4 mm to 1.8 mm and 0 to 4 N that also could be used in the force–displacement simultaneous assessment. However, this region was not used in this work, since the other region (from 4 N to 11 N) represents a higher measurement range. The optical power attenuation analysis was performed to obtain additional data on displacement and force with a single FBG. If a measurement of displacement or force is desired, i.e., only one of the two variables, the optical attenuation analysis is unnecessary, since higher correlation is found in the wavelength shift as a function of the force or displacement. However, if both variables (force and displacement) need to be simultaneously measured with a single FBG, we need additional spectral data to obtain a system with two data and two variables. Thus, the optical attenuation results presented in Figure 5 are used only when the force and displacement need to be simultaneously measured with a single FBG.

Comparing the R^2^ of the wavelength regression as a function of each parameter, there was a higher correlation of the wavelength shift with the displacement, which indicates that the strain transmission between the diaphragm and optical fiber has a higher correlation to the displacement applied or induced in the material than the force. Similar analysis was performed with the reflected optical power, where the highest R^2^ was found in the regression with force, which indicates a higher correlation between the force and optical power variation. Nevertheless, it is worth noting that such a correlation is for a limited range and the difference between the R^2^ of displacement (0.965) and force (0.986) was smaller than the differences of displacement and force (0.998 and 0.966, respectively) when the wavelength shift was analyzed. Thus, we can assume that displacement has a higher correlation with the FBG response than the force, since the force (stress) is related to the displacement (strain) transmission in the diaphragm, related to its mechanical properties.

The correlation between the spectral features and applied loadings as well as the different sensitivities found for each case indicate the possibility of simultaneous assessment of force and displacement using both features in the spectral response, i.e., the optical power and wavelength shift regressions as a function of displacement and force can be applied in Equation (4) to simultaneously estimate both parameters.

(4) $$\left[\begin{array}{c} \Delta \lambda_{B}\\ \Delta P \end{array}\right]=\left[\begin{array}{cc} K_{x,\lambda } & K_{F,\lambda }\\ K_{x,P} & K_{F,P} \end{array}\right]\left[\begin{array}{c} \Delta x\\ \Delta F \end{array}\right]$$

where Δ*λ_B_* and Δ*P* are the wavelength shift and optical power variation, respectively. Similarly, Δ*x* and Δ*F* are the displacement and force variations, respectively. The wavelength shift sensitivities to displacement and force are represented as *K_x,λ_* and *K_F,λ_*, respectively. The parameter *K_x,P_* represents the optical power sensitivity to displacement, whereas *K_F,P_* represents the optical power sensitivity as a function of the applied force.

**Figure 6:**
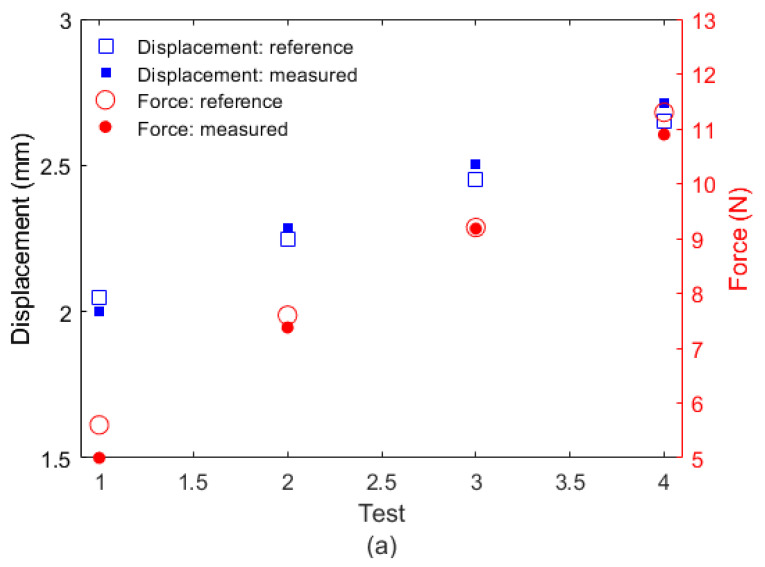

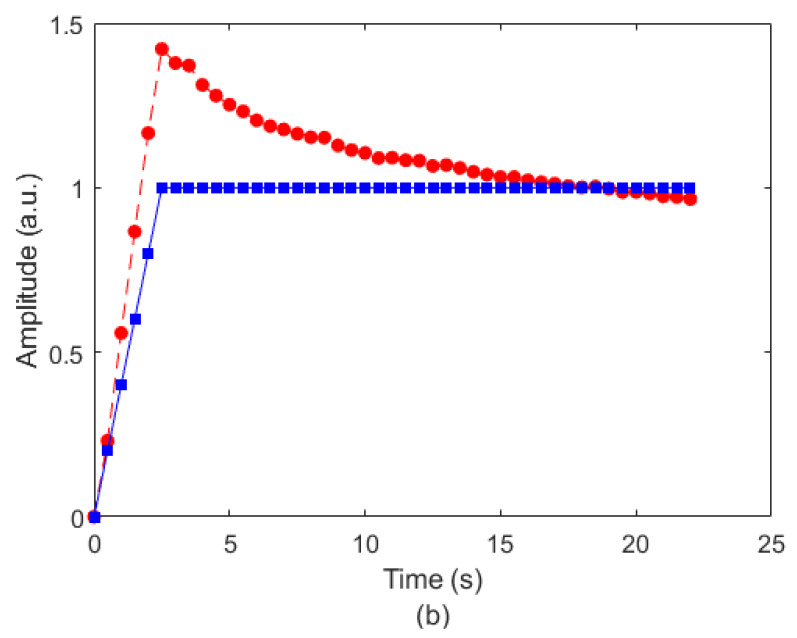
(**a**) Force and displacement estimation with FBG wavelength shift and reflected optical power; (**b**) force as a function of time for a step displacement input.

Following the force and displacements characterizations, the simultaneous assessment of both parameters was performed by applying the sensitivity parameters in Equation (4) and with the spectral features, reflected optical power, and wavelength shift in the regions at which there was a linear regression of both wavelength shift and reflected optical power as a function of force and displacement. The matrix determinant was calculated, since for the displacement and force estimation, the sensitivity matrix should be inverted. In this case, the matrix determinant was −0.12, which indicates that the matrix had an inverse and shows that there was no linear dependency between the matrix’s elements. Figure 6a shows the force and displacement estimation results using the proposed approach, where the displacement and force sensitivities were applied in Equation (4) within the linear range of both parameters. The results were compared with the parameters measured by the displacement and force sensors (strain gauge) of the experimental setup (see Figure 2). The results indicated a good agreement between the estimated results (from the FBG response) and the reference sensors due to the small root mean squared error (RMSE) of 0.05 mm and 0.37 N for displacement and force, respectively.

It is worth mentioning that the force application leads to a displacement on the material (and vice-versa) due to the stress–strain relations of the material. Thus, the analysis of the sensor’s response to displacement and force can enable the assessment of the delay (or phase shift) between the stress and strain as well as the creep behavior. In this case, a step displacement was applied, where force and displacement were normalized as shown in Figure 6b. The results showed a shift between force and displacement, which can be correlated with creep and stress relaxation responses in viscoelastic materials, as presented in ref. [32]. Such relaxation in the force response is transmitted to the optical fiber and indicates the possibility of using FBGs as a tool for in situ material analysis.

In Figure 6b, the results are normalized with respect to the values after a state close to a steady-state is reached, i.e., the mean of the values after 10 s. For this reason, the maximum value for the displacement was 1, whereas the maximum value of the force was higher than 1 due to the material viscoelasticity with a creep/relaxation behavior related to the rubber in which the FBG was embedded. In this case, the displacement applied in the sensor can directly be measured either by the displacement sensors in the machine or by the FBG sensors using Equation (4). Moreover, the force was also measured by the FBG sensors, which show the feasibility of measuring both variables simultaneously with the additional possibility of estimating the creep/relaxation effects of viscoelastic materials, such as plastics and rubbers.

## 5. Conclusions

This paper presented a diaphragm-embedded FBG sensor for force and displacement analysis. A numerical analysis considering a linear elastic material indicated a linear relation between the strain in the fiber with displacement and force. The experimental analysis was performed by embedding the FBG sensor in a rubber diaphragm, where the material’s properties could lead to different relations between stress and strain applied on the diaphragm material. Thus, there were differences in the sensor responses to displacement and force. To that extent, two sets of experimental tests were performed with displacement control and with force control. In this case, the wavelength shift and reflected optical power were analyzed as a function of the displacement and force. The results showed the differences between the sensitivities of displacement and force, where the highest correlation (R^2^ of 0.998) was obtained from the displacement tests, which indicated that there was a higher correlation between the strain applied on the diaphragm and the sensor response. In contrast, the optical power variation was linear only on a limited force and displacement range. In this linear range, it is possible to simultaneously estimate the force and displacement with RMSE of 0.37 N and 0.05 mm, respectively, by analyzing the FBG wavelength shift and optical power variation. In addition, the results also showed the possibility of estimating the material properties, including phase delay, creep, and relaxation in viscoelastic materials using FBG sensors. Therefore, the results obtained in this paper show a new possibility in FBG sensing as the sensor can be directly applied on in situ material analysis as well as simultaneous assessment of force and displacement. Future works include the use of the proposed approach in materials of different natures and types such as metals, ceramics, thermosetting polymers, and even some high-viscosity fluids.
